# Association of Pregnancy Intention with Parenting Difficulty in Fukushima, Japan

**DOI:** 10.2188/jea.15.244

**Published:** 2005-11-07

**Authors:** Aya Goto, Seiji Yasumura, Junko Yabe, Yukiko Anazawa, Yuko Hashimoto

**Affiliations:** 1 Department of Public Health, Fukushima Medical University School of Medicine.; 2 Public Health Section, Social Health and Welfare Department, Sukagawa City, Fukushima.

**Keywords:** Pregnancy, Unwanted, Child Rearing, Parenting, Japan

## Abstract

BACKGROUND: Our prior study revealed that nearly half of the Japanese women between the ages of 35 and 49 years experience an unintended pregnancy, many of which are carried to term. The present study is intended to investigate the association of the intention to become pregnant with parenting difficulty after birth.

METHODS: We distributed self-administered questionnaires to mothers of 317 randomly selected children aged 3 to 18 months who resided in Sukagawa City, Fukushima. The extent to which the unintended pregnancy is associated with the risk of negative attitude in parenting was examined by using multiple logistic regression analysis.

RESULTS: The response rate was 69% and the proportion of births, the outcome of an unintended pregnancy, 22%. When the pregnancy was unintended, the mother may not deny her feelings toward child abuse (odds ratio [OR] = 5.2). She was unlikely to have discussion about child rearing with her husband (OR=3.1) or family (OR=3.3); or the husband rarely participate in child rearing (OR=1.9).

CONCLUSION: To improve the child rearing environment, these findings provide preliminary evidence to underscore the importance of pregnancy planning and providing services to augment the parenting skills of couples who have an unintended pregnancy. A follow-up of pregnant women is currently underway to examine prospectively the effects on child rearing of one’s intention to become pregnant.

Our previous study revealed that 46% of Japanese women aged 35-49 years had an unintended pregnancy, 41% of which ended in a live birth.^1^ When carried to term, it is associated with the mother’s negative antenatal health behavior, poor child health combined with inadequate psychosocial development and problematic child-parent relations.^2^ In Japan, not much attention has been paid to the influences and consequences of an unintended pregnancy. This is the second in a series that explores how the intention to become pregnant associates with parenting difficulty in a city in Fukushima Prefecture.

## METHODS

This cross-sectional study was conducted in Sukagawa City, Fukushima, as one of the health activities run by the social health and welfare department of the city. The study sampled mothers of children aged 3 to 18 months who resided there. From a residence registry with a sampling ratio of 33%, 317 children were randomly selected. The self-administered questionnaires were distributed and collected from their mothers via mail in July 2002 (a reminder was distributed to increase the response rate). To ensure anonymity, the subjects were asked not to identify themselves in the questionnaires.

The questionnaire consisted of three sections: socio-demographic background, ante- and post-natal health behavior and child rearing. A question on pregnancy intention was designed based on a definition used in the National Survey of Family Growth (NSFG) developed in the United States.^3^ The same question was used in our previous study,^1^ after its test-retest reliability was confirmed in a pilot project.^4^ Another question was added here to explore further a woman’s understanding of her intention to become pregnant. Each mother rated her feelings about the pregnancy on a 10-point scale, as in the NSFG.^3^ The scale ranged from 1 = “very unhappy” to 10 = “very happy.” Five questions regarding child rearing were taken from the National Child Health Survey^5^ because they had been adopted as indicators in The 21st Century Sukoyaka (Healthy and Happy) Family, a nationwide agenda for the promotion of maternal and child health in Japan.^6^

All data were entered into a computer and analyzed by using statistical software STATA^®^ version 8 for Windows (Stata Corporation, College Station, TX). The extent to which unintended pregnancies are associated with a higher risk of negative parenting was examined, using multiple logistic regression analysis, while controlling for the following seven factors: mother’s age and occupation, father’s occupation, child’s age and birth weight, support from grandparents (either they live together with grandchildren or look after grandchildren during the day), and living with siblings. These factors are known to relate to parenting difficulty,^7^^-^^10^ which increases as the child ages.^7^ Additionally, living with a father was put into the analysis of his participation in child rearing as a controlling factor.

## RESULTS

Of the 317 randomly selected subjects, 217 returned the questionnaires (response rate = 69%). Twenty were eliminated from the analysis because they had been completed by persons other than the mothers or the pregnancy intention was unknown. Subsequently 197 were analyzed.

The median age of the 197 children in question was 10.5 months (min. = 3, max. = 19); 51% were male; 10 were born with low birth weight; and 52% lived with siblings. The median age of the mothers was 29 (min. = 18, max. = 40); 58% were housewives; and 43% lived with their parents or had them look after the children. Twelve fathers did not live with their children; and 83% were office workers. The proportion of births that were the results of unintended pregnancies was 22%. The feeling about pregnancy was significantly more negative for unintended (median, 8) than intended (median, 10) pregnancy (Wilcoxon rank sum test, p<0.01).

The association between the intention to become pregnant and parenting difficulty is shown in Table 1. An unintended pregnancy was associated with a higher risk of not denying the feeling of abusing a child (odds ratio [OR] =5.2), not discussing child rearing with one’s husband (OR=3.1) or family (OR=3.3), and the husband’s infrequent participation in child rearing (OR=1.9).

**Table 1.** Association of pregnancy intention with parenting difficulty

	N (%)^†^		

	Intended	Unintended	Odds ratio^‡^
	(N=153)	(N=44)	(95% CI)
Have confidence in child rearing (No or not sure)	91 (59.9)	29 (65.9)	1.6 (0.7- 3.7)
Feel I am abusing my child (Yes or not sure)	10 ( 6.6)	7 (15.9)	5.2 (1.3-20.8) *
Have time to interact with child in relaxed mood (No or not sure) Discuss about child rearing with:	38 (25.0)	13 (29.6)	1.1 (0.4- 2.6)
Discuss about child rearing with:			
Husband (No)	35 (22.9)	18 (40.9)	3.1 (1.3- 7.7) *
Family members (No)	30 (19.6)	14 (31.8)	3.3 (1.4- 8.0) *
Think the baby’s father is cooperative in child rearing (Not very much or not at all)	64 (43.0)	26 (60.5)	1.9 (0.9- 4.2) ^#^

† : The denominator of the proportion (in parentheses) is the total number of each group indicated in the top row.

‡ : The odds ratio was calculated by using women who intended to become pregnant as a reference group and adjusted by logistic regression for seven factors: mother’s age and occupation, father’s occupation, child’s age and birth weight, support from grandparents and living with siblings. Living with father was added for the analysis of father’s participation in child rearing.

# : p<0.1, *: p<0.05

CI: confidence interval

## DISCUSSION

One advantage of this study is a new question that asks a woman to rate her feelings about pregnancy using a 10-point scale. As expected, the median score was higher when pregnancy was intended. Comparing the data from previous research on the proportion of births as a consequence of an unintended pregnancy, our result was only slightly lower than one study conducted in Japan, using the NSFG’s pregnancy intention classification (26%).^11^ The results confirm the internal and external validity of our pregnancy intention question.

When interpreting the results of this study, three limitations were found in addition to the small sample size. First, the pregnancy intention was questioned retrospectively. There could be a problem with the recall bias, because a woman’s feelings toward past pregnancies may be influenced by current social circumstances and may change over time. We tried to minimize the bias by targeting mothers who had delivered within 2 years before the survey. Second, questions regarding parenting were limited to those asking for the mother’s subjective opinions, while other measures to detect potential child maltreatment cases were not included. The association of pregnancy intention with child maltreatment should be further explored. Third, a question regarding one’s socio-economic status, considered to be highly sensitive, was not included in this survey, which was conducted as part of the city’s health promoting activities. Father’s occupation thus was entered into the multivariate analysis as its substitute. Including socio-economic indicators in the analysis might have shown different results.

Although the findings are preliminary, this study shows a notable association between pregnancy intention and parenting difficulty. It was found that an unintended pregnancy was associated with a higher risk of fostering a feeling of situational abusive behavior, which may lead to actual child maltreatment. When one faces child rearing without planning in the course of life, it can disrupt their lives and contribute to inadequate child care.^2^ The current data also showed that a higher proportion of mothers who had an unintended pregnancy reported poor support from their respective husbands. It is known that an unintended pregnancy can place a strain on the parental relationship and be associated with the father’s ability to provide positive parenting support.^2^ Along with little support from their partners, the mothers of an unintended pregnancy discussed less about child rearing with their families. The data illustrate that those women who have undergone an unintended pregnancy tend to suffer alone from the burden of parenting.

One of the four major objectives of “The 21st Century Sukoyaka (Healthy and Happy) Family” campaign is to promote the healthy psychological development of children and to alleviate the parents’ anxiety about child rearing.^6^ To improve the child rearing environment and prevent parenting difficulties and child maltreatment, the current findings provide preliminary evidence to underscore the importance of pregnancy planning and offering services to increase the parenting skills of those couples who had had an unintended pregnancy. We are currently conducting a follow-up survey of pregnant women to examine prospectively the effects of pregnancy intentions on child rearing.
